# Umbilical cord blood acid-base analysis and the development of significant hyperbilirubinemia in near-term and term newborns: a cohort study

**DOI:** 10.1186/s13052-017-0382-8

**Published:** 2017-08-04

**Authors:** Vincenzo Zanardo, Federico de Luca, Alphonse K. Simbi, Matteo Parotto, Pietro Guerrini, Gianluca Straface

**Affiliations:** 1 Division of Perinatal Medicine, Policlinico Abano Terme, Piazza Colombo 1, 35031 Abano Terme, Italy; 2 Division of Social Statistics and Demography, University of Southampton, Southampton, UK; 3 Department of Anesthesia, University of Toronto, Toronto, ON Canada

**Keywords:** Cord blood base deficit, Early discharge, Hyperbilirubinemia risk, Jaundice, Newborn

## Abstract

**Background:**

The recognition, follow-up, and early treatment of neonatal jaundice has become more difficult, since the earlier discharge of newborns from hospitals has become common practice. Since intrapartum hypoxic stress has been pointed as predisposing factor for the occurrence of hyperbilirubinemia risk, we tested the association with the cord blood acid-base index tests.

**Methods:**

A cohort of healthy term and near-term newborns underwent umbilical cord hemogasanalysis at birth and capillary heel total serum bilirubin (TSB) pre-discharge, scheduled at 36 h of life, to define the risk of significant hyperbilirubinemia, defined as >9 mg/dL TSB level, ≥ 75th percentile on nomogram of Bhutani et al.

**Results:**

It was found that among 537 studied neonates, 133 (24.8%) had pre-discharge TSB levels of >9 mg/dL. When the cord blood gas analysis index tests were compared, their acidemia levels were significantly higher than those of neonates with normal TSB levels: HCO_3_^−^ (20.71 ± 2.37 vs. 21.29 ± 2.25 mEq/L, *p* < 0.010), base deficit (−3.52 ± 3.188 vs. -2.68 ± 3.266 mEq/L, *p* < 0.010), and lactacidemia (3.84 ± 1.864 vs. 3.39 ± 1.737 mEq/L, *p* < 0.012), respectively. However, logistic regression analysis showed that base deficit was the strongest index of the pre-discharge hyperbilirubinemia risk (OR, 95% CI 0.593; 0.411–0.856), and the hyperbilirubinemia risk increased by 40% with the decrease of 1 mEq/L of base deficit.

**Conclusions:**

Umbilical cord blood acidemia and lactacidemia are significant indexes of adaptive mechanisms at birth. The base deficit provides the strongest association with future development of high bilirubin on an hour specific bilirubin nomogram generating risk stratification score in term and near-term neonates.

## Background

Hyperbilirubinemia is a frequently encountered problem during the first week of life in most healthy newborns due to elevated levels of unconjugated bilirubin, the final product of heme degradation generated by increased breakdown of fetal erythrocytes, deficient human serum albumin transport to the liver, inefficient conjugation, and excretion [1, 2]. In fact, although up to 60% of term newborns have clinical jaundice in the first week of life, few have very high unconjugated bilirubin levels and significant underlying disease, such as hemolytic disease, metabolic and endocrine disorders, anatomic abnormalities of the liver, infections, and asphyxia, all enhancing the risk of developing acute encephalopathy or disabling neurological auditory and motor sequelae in kernicterus [3].

Recently, the recognition, follow-up, and early treatment of neonatal jaundice has become more difficult, since the earlier discharge of newborns from hospitals has become a common practice because of medical and social reasons and economic constraints [4] and it is significant that the most common cause for readmission to the hospital during the early neonatal period is hyperbilirubinemia [5]. To reduce bilirubin induced neurological dysfunction incidence, the American Academy of Pediatrics recommended that all newborn infants be assessed before discharge for the risk of developing significant neonatal hyperbilirubinemia [6]. These Guidelines suggest two risk-assessment options, used individually or in combination: pre-discharge measurement of the bilirubin level and assessment of clinical maternal, infant, and delivery risk factors known to be associated with neonatal hyperbilirubinemia [7–9].

However, a few studies have formalized the clinical risk factor assessment process by developing scoring systems that incorporate the weighted contribution of multiple risk factors at once. In addition, the accuracy of clinical risk factor assessment has been attenuated by omission of test useful to measure important clinical predictors and/or lack of hour-specific bilirubin nomogram to calculate an infant’s bilirubin percentile with respect to age in hours, to support the pre-discharge hyperbilirubinemia “risk zone” [10].

Hypoxemia, perinatal asphyxia, as well as acidosis have been pointed as predisposing risk factors for the occurrence of significant unconjugated hyperbilirubinemia and the need of phototherapy and, by itself, can abide the risk of brain damage [11–14], In accordance with the Literature, although in many cases hypoxemia and acidosis seem to be well tolerated by newborns [15], a more pronounced hypoxic insult can bear the risk of cerebral damage and/or affect the bilirubin/albumin ratio for binding and the ability of properly process bilirubin to the direct form, increasing the rate of occurrence of unconjugated bilirubin encephalopathy [16]. Although an interdisciplinary approach could help put neonatal care on a different basis, the role played by hypoxia or acidosis during unconjugated bilirubin brain damage cytotoxicity has been scarcely investigated, and the few available in vitro studies used a model of simultaneous association of both conditions [17].

On the basis of this background, the aim of our study was twofold: (i) investigate in an non-interventional way the value of the acidemia index tests in cord blood gas analysis for the occurrence of pre-discharge TSB risk, calculated by the infant’s bilirubin percentile “risk zone”, plotted on the hour-specific bilirubin nomogram of Bhutani et al. [18, 19]; (ii) examine whether lactacidemia, simultaneously analysed with the hemogasanalysis in cord blood samples, may be another complementary index.

## Methods

### Setting

This study was performed at the Division of Perinatal Medicine of Policlinico Abano Terme, Abano Terne (Italy) during a seven-month period ended on December 2015, after Institutional Review Board (Policlinico Abano Terme) approval was obtained.

### Participants

All healthy full-term and near-term (≥34 weeks of gestation) newborns delivered by vaginal birth or caesarean section during this period were prospectively enrolled in the study. Informed consent was obtained from all parents of the newborns enrolled in the study.

Neonates were eligible if an umbilical artery blood sample for blood gas analysis was correctly obtained at birth and if they had no evidence of severe clinical diseases or any other complications; no evidence of congenital or chromosomal anomalies, intrauterine growth restriction (estimated fetal weight < 10th percentile) or NICU admission. Exclusion criteria were also Rh or major ABO isoimmunisation indexed by a positive direct antiglobulin test, drugs administered to the infants, except for 1 mg vitamin K (Konakion®) intramuscularly soon after birth.

### Study design

Umbilical artery hemo gas analysis and complementary test lactacidemia were measured in the blood drawn from the umbilical arteries immediately after delivery by ABL90 FLEX Radiometer® analyzer (Radiometer®, Copenhagen, Denmark).

All deliveries were assisted by certified midwives. A standard assistance delivery protocol was implemented in all cases, and it included: expectant management during the transition phase; free maternal position during the expulsion of the fetus; avoidance of any unnecessary manual intervention by the providers; optimization of pushing efforts to coincide with maternal urge to push; restriction of operative delivery by vacuum extractor and/or episiotomy to limited indications (I.e., fetal distress). Mothers who delivered vaginally were not under any food or liquid restrictions. Mothers who underwent caesarean delivery were fasting at least 6 h before the caesarean section and by delivery room protocol, received parenteral Ringer lactate solution prior to the anesthesia procedure. The cord-clamping timing was within the first 60 s after birth and sampling techniques used in the vaginally and caesarean delivery groups were similar and previously reported [20].

The study was carried out within the context of the hospital’s ordinary care protocol. In accordance with the hospital’s standard practice, following an uneventful delivery, infants are placed on the mother’s chest for about 15 min during which time the midwife assists with the first suckling episode. Infants are then dried, receive umbilical care, and are weighed before their first bath. During the subsequent 2 days in our ward, newborns room-in with their mothers, who are encouraged to demand-feed them (with no more than 3-h interfeeding intervals). The infants receive complementary formula milk if breast milk intake is judged insufficient by the midwives.

No prophylactic intervention for hyperbilirubinemia was employed. Environmental lighting was constant during the study period.

### Data collection

After obtaining parental informed consent, TSB, hematocrit, and body weight measurements were subsequently made at 36 h of life. Bilirubin values obtained before discharge were plotted on an hour-specific bilirubin nomogram [18, 19] to calculate an infant’s bilirubin percentile with respect to age in hours. The pre-discharge bilirubin “risk zone” (0–40th, 41st to 75th, 76th to 95th, and >95th percentile corresponding with low, low-intermediate, high-intermediate, and high-risk zones on the hour-specific bilirubin nomogram) has been shown to be a strong predictor of subsequent risk of hyperbilirubinemia [21] for jaundiced at term and near term newborn infants. Accordingly, newborn infants with total serum bilirubin (TSB) levels of >9 mg/dL at 36 h of life, the 75th percentile cut-off of TSB nomogram of Bhutani et al. [18, 19], were defined to have significant hyperbilirubinemia. TSB was measured on capillary blood (TOTAL BILI, Officina Biomedica, Italy) at 36 h of life and then every 12–24 h when clinically indicated. Newborn babies needing phototherapy [18] were discharged after a TSB decrease at two consecutive samples. Mother-infant clinical and anthropometrical data were obtained from the hospital birth records. The information collected through the data forms was entered in a database by different operators using the double-entry technique. These operators also checked for and validated inconsistencies. Case charts were reviewed whenever a flaw was detected.

### Statistical analysis

Continuous variables are presented as means and standard deviations (SD), categorical data are presented as numbers and percentages. Multivariate logistic regression was used for testing variables’ predictivity of bilirubin levels >9 mg/dL at 36 h of life. We first adapted to the data a model which included all the variables which we had available in order to predict the risk of bilirubin levels >9 mg/dL (complete model). Then, with a stepwise selection, we excluded from the model all those variables which did not increase significantly the predictivity of the model (pH, PCO_2_, lactacidemia, absolute post-birth weight loss, delayed meconium emission, and haematocrit. In addition, we included in the model related important control variables, regardless of their statistical significance: gender, gestational age, birth weight, and whether a caesarean delivery was necessary. Significance was tested using the standard Wald test, and a *p* value below 0.05 was considered statistically significant. Data analysis was performed with STATA for Windows statistical package (version 11).

## Results

A total of 544 newborns were initially enrolled in the study, but 7 of these were excluded during the study because of various diagnoses, such as duodenal atresia (1 neonate), direct hyperbilirubinemia (1 neonate), and infection or sepsis (2 neonates), or because some (3 neonates) of the parents did not want to continue participating in the study refusing metabolic screenings.

In particular, we found that 133 (24.8%) of 537 newborns screened had TSB levels of >9 mg/dL at 36 h of life, corresponding to >75th high-intermediate ‘risk zone’, with a TSB mean value significantly higher (10.04 ± 0.798 vs. 6.92 ± 1.612 mg/dL, *p* < 0.001).

Neonates with pre-discharge TSB ≤9 or >9 mg/dL were comparable for gestational age, birth eight, mean postnatal body weight decrease, and sex.

However, these neonates at risk of hyperbilirubinemia, were significantly more frequently vaginally delivered and presented significantly higher hematocrit values both at birth and pre-discharge. Conversely, they presented significantly higher acidemia (HCO_3_^−^ and base deficit) and lactacidemia index texts in cord blood gas analysis at birth (Table 1).

**Table 1:** Hemogasanalysis components levels in neonates according to significant hyperbilirubinemia, total serum bilirubin >9 mg/dl at 36 h of life

	Total Serum Bilirubin at 36 h		
	≤9 mg/dL	>9 mg/dL	p
Group size, %	404 (75.2%)	133 (24.8%)	
Bilirubin at 36 h, mg%	6.92 (1.612)	10.04 (0.798)	<0.001
Cesarean delivery rate, %	27.73 (44.31)	14.29 (35.13)	0.001
Gestational age, weeks	39 (1.18)	39 (1.33)	0.212
Female, %	53.22 (49.96)	45.86 (50.02)	0.142
Birth weight, g	3320 (436)	3340 (419)	0.643
Body weight at 36 h, g	3112 (413)	3125 (397)	0.753
Delta weight decrease, g	−207 (66)	−211 (64)	0.574
Hematocrit at birth, %	49.44 (4.75)	51.65 (5.16)	<0.001
Hematocrit at 36 h, %	52.10 (6.83)	55.72 (6.58)	<0.001
pH, units	7.34 (0.076)	7.34 (0.079)	0.736
PaCO_2_, mmHg	43.10 (9.47)	41.45 (8.54)	0.076
PaO_2_, mmHg	23.93 (9.70)	23.86 (7.78)	0.938
HCO_3_^−^, mEq/L	21.29 (2.25)	20.71 (2.37)	0.010
Base Deficit, mEq/L	−2.68 (3.266)	−3.52 (3.188)	0.010
Lactacidemia, mEq/L	3.39 (1.737)	3.84 (1.864)	0.012

Mean (± Standard Deviation, SD) or Percentage if not otherwise stated

*P* < 0.05, Statistical significance by Student *T* test

Logistic regression analysis (Odds Ratio; 95% Confidence Interval) showed that gestational age and birth weight were significantly associated with the risk of TSB >9 mg% at 36 h of life. Noteworthy, among the cord blood hemogasanalysis index texts, only PaO_2,_ HCO_3_^−^, and base deficit were significantly related to the risk of TSB >9 mg%. Among these, base deficit showed the strongest association with pre-discharge hyperbilirubinemia risk, revealing that the hyperbilirubinemia risk increased by 40% with the decrease of 1 mEq/L of base deficit. Finally, also lactacidemia showed a statistically significant association with hyperbilirubin risk (Table 2).

**Table 2:** Logistic regression of hyperbilirubinemia (>9 mg%): estimates of Odds Ratios and their 95% Confidence Interval

Risk factors	OR	95% CI
Gestational age	0.744 ***	0.606–0.914
Birth weight	1.059 *	1.001–1.121
PaO_2_	0.950 *	0.908–0.994
HCO_3_^−^	1.929 **	1.172–3.174
Base deficit	0.593 ***	0.411–0.856
Hematocrit	1.127 ***	1.073–1.184
Cesarean delivery	0.638	0.341–1.195
Female	0.822	0.539–1.256

*p 0.01 ≤ − ≤0.05

** p 0.005 ≤ − ≤ 0.01

***p ≤ 0.005

## Discussion

The main finding of this study is that acidemia components of cord artery blood gas analysis at birth are significantly associated with bilirubin levels at 36 h of life and that among acidemia components and other complementary tests, base deficit provides the most significant association with the pre discharge hyperbilirubin risk,’ > 9 mg/%, representing the 75th percentile cut-off on total serum bilirubin values ‘risk zone’, plotted on the TSB hour-specific nomogram of Bhutani et al. [18, 19] for jaundiced term and near-term newborn infants.

Use of umbilical cord blood gas analysis in the assessment of the newborn risk of hyperbilirubinemia has not been previously investigated, although classical studies supported the assumption that hypoxemia, low Apgar score, as well as acidosis may have been pointed as predisposing risk factors for cerebral damage and the occurrence of significant unconjugated hyperbilirubinemia and the need of phototherapy [16]. However, the role played by hypoxia or hypoxia–ischemia during unconjugated hyperbilirubinemia cytotoxicity has been scarcely investigated. Furthermore, the few available in vitro studies used a model of simultaneous association of both conditions [20].

The interpretation of a potential etiopathogenetic role of cord blood base deficit in determining pre-discharge hyperbilirubinemia risk is complex, and involves consideration of an array of maternal, infant, and delivery factors known to be associated with neonatal hyperbilirubinemia. Findings in previous studies suggested that the base deficit in umbilical blood may mirror the duration of hypoxia [21]. Thus, calculation of the base deficit should be the best way of quantifying a change of buffer balance because it takes in to consideration both the carbon and non-carbon buffering systems [22]. Hypoxemia, acidemia and hypoalbuminemia were significantly associated with the need for phototherapy, which is in accordance with the literature [23, 24]. Conditions such as acidosis and hypoxia at birth can affect the bilirubin/albumin ratio for binding, whereas the presence of any type of liver disease or a metabolic or enzyme disorder will also affect the ability of the body to properly process bilirubin to the direct form to allow for excretion [25]. Perinatal asphyxia, as well as prematurity, acidosis and respiratory distress, have been also pointed as predisposing risk factors for the occurrence of unconjugated bilirubin encephalopathy [26]. Therefore, although both conditions can occur simultaneously, it is important to consider that neonatal hyperbilirubinemia can follow a birth hypoxia state and that base excess may provide the most significant estimate about the pre-discharge hyperbilirubin risk.

This study has however, some limitations. Although the target population for our study to which the results would be generalized was all term and near-term neonates we excluded from the analysis the asphyxiated or premature neonates at higher risk of acidemia admitted to special care baby unit. The critical limits of umbilical artery blood gas values also vary widely in the literature [23] and we lack information about the modality of labor induction and/or use of oxytocin, and the blinding of some diagnostic tests such as cardiotocography or STAN [27], each exerting different metabolic stress on the fetus. These considerations did not, however, influence our conclusions, as the study’s primary outcome was umbilical artery blood hemogasanalysis components and significant hyperbilirubinemia risk at 36 h of life, defined according to Bhutani nomogram.

## Conclusions

In summary, hypoxemia, perinatal asphyxia, as well as acidosis have been pointed as predisposing risk factors for the occurrence of significant unconjugated hyperbilirubinemia in neonates. Investigating the relationship between umbilical cord blood acid-base indexes and the development of significant hyperbilirubinemia in a cohost of near-term and term, healthy newborn infants, it was found that, among acidemia indexes inclusive of lactacidemia, base deficit had the strongest association with pre-discharge bilirubin risk, plotted on the hour-specific bilirubin nomogram of Bhutani et al. [18, 19].
